# Decision Rules Construction: Algorithm Based on EAV Model

**DOI:** 10.3390/e23010014

**Published:** 2020-12-24

**Authors:** Krzysztof Żabiński, Beata Zielosko

**Affiliations:** Institute of Computer Science, Faculty of Science and Technology, University of Silesia in Katowice, Będzińska 39, 41-200 Sosnowiec, Poland; krzysztof.kamil.zabinski@gmail.com

**Keywords:** decision rules, classification, length, support, dynamic programming approach, entity–attribute–value model

## Abstract

In the paper, an approach for decision rules construction is proposed. It is studied from the point of view of the supervised machine learning task, i.e., classification, and from the point of view of knowledge representation. Generated rules provide comparable classification results to the dynamic programming approach for optimization of decision rules relative to length or support. However, the proposed algorithm is based on transformation of decision table into entity–attribute–value (EAV) format. Additionally, standard deviation function for computation of averages’ values of attributes in particular decision classes was introduced. It allows to select from the whole set of attributes only these which provide the highest degree of information about the decision. Construction of decision rules is performed based on idea of partitioning of a decision table into corresponding subtables. In opposite to dynamic programming approach, not all attributes need to be taken into account but only these with the highest values of standard deviation per decision classes. Consequently, the proposed solution is more time efficient because of lower computational complexity. In the framework of experimental results, support and length of decision rules were computed and compared with the values of optimal rules. The classification error for data sets from UCI Machine Learning Repository was also obtained and compared with the ones for dynamic programming approach. Performed experiments show that constructed rules are not far from the optimal ones and classification results are comparable to these obtained in the framework of the dynamic programming extension.

## 1. Introduction

Currently, data amounts grow constantly and uncontrollably, practically in every domain of life. It results in the demand for efficient and fast methods of their analysis. Generally speaking, raw data occupy more and more disk space, but without converting them to any kind of useful knowledge, they are just redundant. The question is then how to derive knowledge from raw data. Data mining as a scientific discipline tries to answer it. This domain has been developed for many years already; as a result, there are plenty of already existing methods for multiple applications [1,2,3,4]. Nevertheless, with the growth of data amounts, the existing methods also need to be further developed and new approaches need to be proposed.

The process of knowledge extraction from data sets is called learning. Learning can be basically divided into two subcategories: supervised and unsupervised learning. The main difference is that with supervised learning, there is a supervision of the learning process (which in practice means that data sets are labeled and labels are assigned to each of the item from the set under consideration). As for unsupervised learning, the labeling does not exist, and the task is basically to find any relations between data items. There are also so-called semisupervised learning methods that combine both labeled and unlabeled records being considered [5].

In this article, we study one of the most popular supervised learning methods, i.e., decision rules construction. Such rules are known and popular as a form of knowledge representation used often in the framework of supervised machine learning.

In the general case, decision rules studied in this paper are expressed as follows [6]:

IFconditionsTHENconclusion.

The “IF” part of a rule is known as the rule antecedent (premise part) and it contains one or more conditions (pairs attribute = value) connected by conjunction. The “THEN” part is the rule consequent which present a class label. Based on conditions included in the premise part, the rule assigns class labels to objects. An example would be a rule which predicts when a student enjoy skating:

IF(Temperature=low)AND(Ice_Skating_Rink=yes)THEN(Enjoy_Sport=yes).

Decision rules are popular because of their form which is simple and easy accessible from the point of view of understanding and interpretation of knowledge represented by them. One of the most popular evaluation measure is length, which corresponds to the number of descriptors (pairs attribute=value) on the left-hand side of the rule. Another popular measure is support, which represents the number of objects from the learning set that match the rule. There are also many other indicators for evaluation of decision rules [7,8,9]; however, in this paper, these two are considered.

Construction of short rules with good support is an important task considered in this work. In particular, the choice of short rules is connected with the minimum description length principle [10]: “the best hypothesis for a given set of data is the one that leads to the largest compression of data”. Support allows to discover major patterns in the data. These two measures are interesting from the point of view of knowledge representation and classification.

Unfortunately, the problems of minimization of length and maximization of support of decision rules are NP-hard [11,12,13]. The most part of approaches for decision rules construction, with the exception of brute force, Boolean reasoning, and dynamic programming, cannot guarantee the construction of optimal rules, i.e., rules with minimum length or maximum support. The main drawback of such approaches is limitation of the size of data if the user would like to obtain a solution in some acceptable time. For this reason, authors propose some heuristic, an algorithm for decision rules construction. Generating rules should be enough good from the point of view of knowledge representation and classification.

The paper consists of five main sections. The Introduction, which contains the authors’ contribution, is followed by the Related Works section. Then, in the Materials and Methods section, the proposed approach and main notions, including the entity–attribute–value model, standard deviation function, algorithm for construction of rules and computational complexity analysis, are described. In Section 4, the results of experiments connected mainly with evaluation of constructed decision rules using length, support and classification error are presented. Finally, the conclusions are discussed in Section 5.

### Contribution

In this paper, we propose an algorithm for decision rules construction that belongs to the group of heuristics as it constitutes approximate rules, but which grows from the root of dynamic programming extensions-based approaches. There exist some similarity between these methods connected with partitioning decision table into subtables; however, the main difference is based on selection of attributes and construction of rules which are close to optimal ones. For this reason, the experimental results obtained for the proposed approach were compared with the results known for dynamic programming extensions from the point of view of knowledge discovery and knowledge representation. It was shown that the proposed approach allows us to reduce the number of attributes under consideration even to 60% from the whole set of attributes and obtain classification results comparable to the ones obtained by the dynamic programming extension.

The idea of dynamic programming approach for decision rules optimization is based on partitioning of a decision table into subtables which are created for each value of each conditional attribute. In this way, a directed acyclic graph is obtained which nodes correspond to subtables and edges are labeled by values of attributes. Based on the graph, the so-called irredundant decision rules are described, i.e, rules with minimal length or maximal support. However, if the number of attributes and their values is large, the size of the graph (the number of rows and edges) is huge. Therefore, obtaining an exact solution within a reasonable time is not always achievable.

In the proposed approach, the stage of construction of a graph based on which decision rules are described is omitted. Decision table is partitioned into subtables, but only for the values of the selected attributes. Moreover, to accelerate calculations, the decision table is transformed into the so-called entity–attribute–value model [14]. Selection of attributes and their evaluation is based on the analysis of the spread of their values’ standard deviation for decision classes. If the value of standard deviation is high, there is a high possibility that the attribute can distinguish objects with different decisions. Based on values of selected attributes, corresponding subtables from the input table are created. The process of partitioning of corresponding subtables is finished when all rows in a given subtable have the same class label or all values of selected attributes were considered. Then, decision rules are created basing on corresponding values of selected attributes.

In some authors’ previous work [15,16,17], a modification of the dynamic programming approach for decision rules optimization was proposed; however, the idea was connected with decreasing the size of the graph. Subtables of an input decision table were constructed for one attribute with the minimum number of values, and for the rest of the attributes, the most frequent value of each attribute (value of an attribute attached to the maximum number of rows) was selected. In the presented approach, the idea of selection of attributes is different and construction of the graph is omitted.

## 2. Related Works

There exists a variety of approaches for construction of decision rules. The used approaches depend on the aim for which the rules are constructed. The two main perspectives are knowledge representation and knowledge discovery [18]. Since the aims are different, algorithms for construction of rules with their many modifications are different and quality measures for evaluating of such rules are also different.

Basically, approaches for construction of decision rules can be divided into two categories:

- allowing to obtain exact rules on the data set under consideration,
- generating approximate rules, not perfectly suiting the learning set, but aiming to create rules applicable for general use (these methods will be called heuristics further in this work).

Among the first group, there are algorithms which allow to obtain all decision rules based on exhaustive strategy, there are: the brute-force approach which is applicable to decision tables with a relatively small number of attributes, Boolean reasoning [19], and the dynamic programming approach [20,21] proposed in the framework of rough sets theory [22].

Among the second group of methods, there are popular algorithms based on a sequential covering procedure [23,24,25]. In this case, decision rules are created and added to the set of rules iteratively until all examples from a training set will be covered. Among them, we can distinguish the general-to-specific search methods, e.g., CN2 [26] and PRISM [27] algorithms. In the framework of directional general-to-specific search approach, the AQ family of algorithms can be indicated [28]. The RIPPER algorithm [29] is an example of search methods with pruning. LEM2 belongs to methods based on reduct from rough sets theory [30].

There are also plenty of heuristics which are useful in situations where it is difficult to find an exact solution in some acceptable time. However, such algorithms often produce a suboptimal solution since they cannot avoid local optima. Popular approximate approaches include greedy algorithms [31,32,33] and the whole group of biologically inspired methods, among which the following are worth mentioning: genetic algorithms [34,35,36], ant colony optimization algorithms [37,38], swarm-based approaches [39] and many others as described in [40,41].

The task of constructing a decision rule is similar to the task of finding the features that define entities of some category. Attributes can be considered as functions which mapping a set of objects into a set of attributes’ values. The premise part of a decision rule contains one or more conditions in a form attribute=value. The consequent part of a rule represents a category. For both tasks, there may be a problem how to choose the features that the best describe a given category (concept).

Constructed rules can be considered as patterns that cover many situations and match as many examples as possible, so they should be short according to number of conditions to allows some generalization. However, such rules should also take into account unusual situations, ensuring the correct classification of such objects. Often, the same category is described by more than one rule. Therefore, the set of rules learned from data should not contain contradictory or redundant rules. It should be small such that all rules together cover all examples from learning set and describe a given category in a fairly comprehensive way. As it was mentioned above, there exist plenty of algorithms which use different methods and measures during process of rules construction and choosing attributes that constitute premise part of rules.

## 3. Materials and Methods

In this section, notions corresponding to the proposed approach, the entity–attribute–value model, standard deviation as a distinguishability measure, algorithm for construction of rules and analysis of its computational complexity are presented.

### 3.1. Decision Rules Construction Approach

The three main stages of the proposed decision rules construction approach are the following:

1. Transformation decision table *T* into EAVD model,
2. Calculation of standard deviation based on averages’ attributes values per decision class,
3. Construction of decision rules taking into account selected attributes.

They are discussed in the next sections.

#### 3.1.1. Main Notions

One of the main structure for data representation is the decision table [22]. It is defined as

T=(U,A∪{d}),

where *U* is a nonempty, finite set of objects (rows), $A=\{f_{1},\ldots ,f_{n}\}$ is nonempty, finite set of attributes, $f:U\to V_{f}$ is a function, for any f∈A, $V_{f}$ is the set of values of an attribute *f*. Elements of the set *A* are called conditional attributes and d∉A is a distinguished attribute, called a decision attribute. The decision *d* determines a partition $\left\{Class_{1},\ldots ,Class_{|V_{d}|}\right\}$ of the universe *U*, where $Class_{i}=\left\{x\in U:d\left(x\right)=d_{i}\right\}$ is called the *i*th decision class of *T*, for $1\leq i\leq |V_{d}|$. A minimum decision value that is attached to the maximum number of rows in *T* is called the most common decision for *T*.

The table *T* is called degenerate if *T* is empty or all rows of *T* are labeled with the same decision’s value.

A table obtained from *T* by the removal of some rows is called a subtable of the table *T*. Let *T* be nonempty, $f_{i_{1}},\ldots ,f_{i_{m}}\in \left\{f_{1},\ldots ,f_{n}\right\}$ and $a_{1},\ldots ,a_{m}$ be values of attributes. The subtable of the table *T* that contains only rows that have values $a_{1},\ldots ,a_{m}$ at the intersection with columns $f_{i_{1}},\ldots ,f_{i_{m}}$ is denoted by

$$T^{\prime }=T\left(f_{i_{1}},a_{1}\right)\ldots \left(f_{i_{m}},a_{m}\right).$$

Such nonempty subtable (including the table *T*) is called separable subtable of *T*.

In the paper, decision rule is presented in the following form:

$$\left(f_{i_{1}}=a_{1}\right)\wedge \ldots \wedge \left(f_{i_{m}}=a_{m}\right)\to d=v$$

where *v* is the most common decision for $T^{\prime }$.

The length of the decision rule is the number of conditions (pairs attribute=value) from the left-hand side of rule.

The support of the decision rule is the number of objects from *T* matching the conditional part of the rule and its decision.

#### 3.1.2. Entity–Attribute–Value Model

The proposed approach for construction of decision rules is based on representing decision table in the entity–attribute–value form (abbreviated as EAV). The decision table formed in a way that each object contains a set of conditional attributes’ values and decision (each object occupies one row in the decision table) is converted in a way that each value of attribute constitutes a separate row in the derived EAV table.

EAV form is very convenient for processing large amounts of data. It is mainly due to the fact, that such a form allows to utilize RDBMS (ang. Relational Database Management System) mechanisms directly. The idea to utilize SQL and RDBMS for data mining tasks has known advantages. As SQL is designed to facilitate dealing with large data sets efficiently, it is a natural choice for machine learning related tasks. Additionally, current RDBMSes are well designed to store and retrieve data efficiently and fast. Combining this technological achievement with efficient algorithms can lead to satisfactory level of rule generating system. The idea of SQL-based approach for association rules generation has been introduced in [42] and extended to decision rules construction in [14].

Exemplary EAV table can be created in RDBMS as shown in the Listing 1 (PostgreSQL example).

**Listing 1.** EAV table.
            **CREATE** **TABLE** eav
            (
             id serial **primary** key,
             attribute **character** **varying**,
             **value** **character** **varying**,
             decision **character** **varying**,
             row bigint
            );
		  

In order to have better control on the association of the decision to the attribute value, the EAV form can be extended to contain decision too [43]. Such a format of representation can be denoted as EAVD (entity–attribute–value_with_decision form).

**Figure 1:**
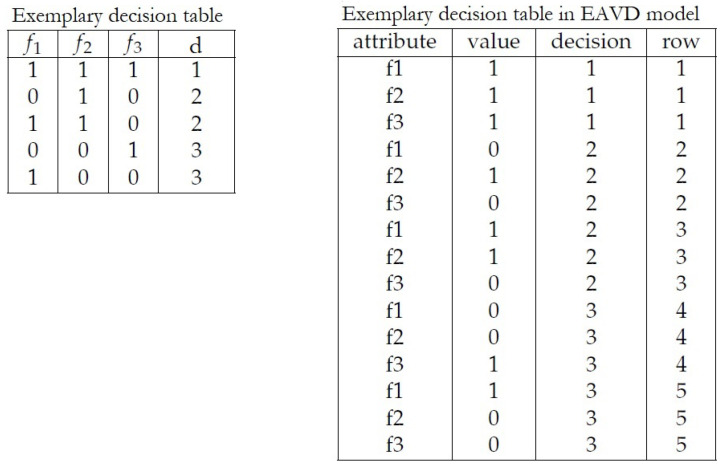
Transformation of decision table into EAVD model.

Figure 1 present exemplary decision table transformed into EAVD model.

#### 3.1.3. Standard Deviation as a Distinguishability Measure

Standard deviation (abbreviated as STD) has been used in this paper as a distinguishability measure. It is based on Bayesian data analysis where attributes and their values are evaluated subject to possible decision classes [44]. We calculated standard deviation of the average values of each decision table attributes grouped per decision values. As attributes in real-life applications often are non-numerical, authors take into consideration numerical equivalents of such attributes. They are simply ordinal numbers according to attributes’ values appearance in the data set under consideration. The higher the standard deviation is, the  greater the diversity among averages values of attributes in particular classes. Such an observation can lead to the conclusion that the higher the STD is, the better the distinguishability between decision classes becomes. As a result, the attributes of high standard deviation should be prioritized when forming decision rules. This forms a ranking of attributes and allows to perform a feature selection step to the rule generation algorithm.

The exemplary SQL code to calculate the mentioned standard deviation is as shown in the Listing 2 (PostgreSQL example).

**Listing 2.** SQL command to calculate standard deviation.
            **SELECT** attribute, STDDEV(average_value) **AS** quality **FROM** (
              **SELECT** e.attribute, e.decision, **AVG**(v.id) **AS** average_value
              **FROM** eav e **JOIN** **values** v **ON** e.**value** = v.**value**
              **GROUP** **BY** attribute, decision
            ) attribute_average_values
            **GROUP** **BY** attribute
            **ORDER** **BY** quality **DESC**
		  

Due to the fact that the proposed algorithm needs to work on any alphanumerical values, there was a need to introduce a dictionary table. Each attribute value needs to be converted into its numerical representative, whilst real values need to be transferred to the proposed dictionary table. The mentioned numerical representatives are subsequent RDBMS tables identifiers (in our approach). The exemplary dictionary table is as shown in the Listing 3.

**Figure 2:**
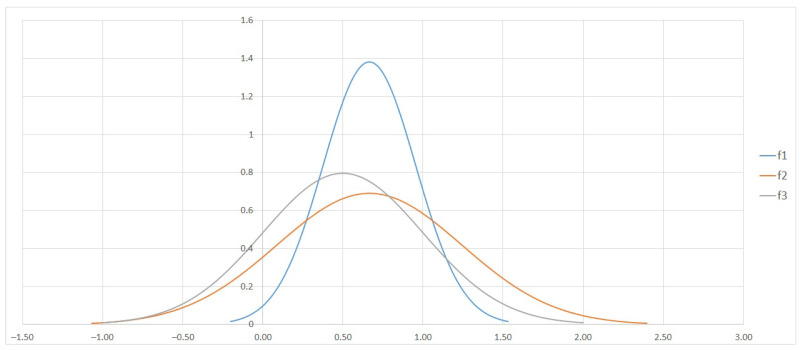
Normal distributions of attributes f1, f2 and f3.

**Listing** **3.** Table containing real values of the given attributes.
            **CREATE** **TABLE** **values**
            (
             id serial **primary** **key**,
             **value** **character** **varying**
           );
		  

In order to better illustrate the distinguishability of attributes based on STD, an exemplary graph has been suggested (Figure 2), for attributes from decision table presented in Figure 1.

It presents normal distributions of attributes among which every has a different STD value. The amplitude of the plots is not important whilst the width of each curve is a direct indicator of the distinguishability level. Basing on such a distribution graph, the conclusion can be make that the ranking of attributes is as follows: f2, f3, f1.

#### 3.1.4. Construction of Decision Rules

The proposed algorithm can be expressed by means of the following pseudo-code (see Algorithm 1). The Algorithm 1 has been designed to deal with discrete attributes. Nevertheless, the is no assumption on numerous nature of attributes. As a result, symbolic attributes need to be converted to numerical representatives. An additional dictionary table has been introduced. It gathers symbolical attributes with their numerical representatives. Having numerical values of attributes obtained, the standard deviation of their average values per decision class can be calculated. These are standard deviations of the average values of each attribute numerical representation for each decision value. The higher the calculated standard deviation, the higher the distinguishability of decision classes basing on the considered attribute. It allows to generate rules basing on only the best attributes from the point of view of distinguishability. The percentage of best attributes that are used for generation need to be chosen empirically and strongly depends on the structure of the data set under consideration. Due to the fact that the attributes are ordered taking into account the distinguishability they offer with respect to the decision values, the generated rules will consist of small number of attributes (making them close to optimal taking into account their length). Creation a ranking of attributes’ basing on STD values introduces a feature selection step. From the point of view of dynamic programming’s algorithm graph construction, the proposed algorithm perform graph pre-pruning, as it omits creation of unnecessary paths that will never be utilized for rules generation.

Having chosen the attributes to be considered, the Algorithm 1 generates rules as described below. Firstly, the set of unique combinations of attributes with values per row in the input decision table needs to be determined. The combinations contain only of the best attributes chosen in the previous step. It is where the row information is also needed. The attributes need to appear in the subsequent combinations ordered descending by the values of previously calculated standard deviations. Nevertheless, the information on decision is not taken into consideration at this stage.

Then, for each attribute combination, the subsequent separable subtables of the input decision table get determined. It makes the algorithm similar to the dynamic programming approach which utilizes partitioning of the decision table into separable subtables. For each of the attribute combinations, the procedure starts by taking the first attribute with its value (it is visible now why the attributes in combinations need to be ordered decreasingly by the previously calculated standard deviations). For this attribute and its value, the separable subtable of input decision table gets determined. After that, it is verified if the subtable is degenerate. If it is in fact degenerate, a decision rule is constructed. It consists of the attribute with its value and the decision in the degenerate table. If the separable subtable is not degenerate, the subsequent attribute from the attribute combination gets chosen and then the subsequent separable subtable gets generated. If it is degenerate, the procedure stops and a decision rule gets generated. The procedure continues until either the subtable is degenerate or all the attributes of a given combination are processed. If all the attributes have been used and the subtable is still not degenerate, a decision rule gets generated basing on all these attributes with their values and the most common decision from the corresponding separable subtable.
**Algorithm 1** Pseudo-code of algorithm generating decision rules for a decision table *T*.**Input:** 
Input decision table *T*, number *p* of best attributes to be taken into consideration.**Output:**  Set of decision rules *R* represent *T* as entity–attribute–value (EAV) form with separate decision; represent each attribute’s value in a discrete numerical form; obtain attributes’ standard deviation per decision class. take *p* number of attributes of largest STD—in a descending order; from *T* in EAV form select sets *v* of unique values (including decision) of attributes grouped per decision table’s rows; **while** there exist sets $v_{i}$ in *v* not marked as processed **do**  generate one-item $v_{i}^{\prime }$ set with initial value from $v_{i}$ which corresponds to creation of separable subtable $T^{\prime }=T\left(f_{i},a_{i}\right)$;   set $v_{i}$ is not processed;
  **while** iterations number < $sizeof(v_{i})$ OR separable subtable is not degenerate **do**   extend $v_{i}^{\prime }$ by supplying it with the subsequent element from $v_{i}$ which corresponds to next partition of $T^{\prime }$;
  **end while**
  generate decision rule basing on the values of attributes from $v_{i}^{\prime }$ (consequent is the most common decision for $T^{\prime }$ corresponding to $v_{i}^{\prime }$);
  supply the set *R* with the newly created rule;
  set $v_{i}$ being processed.
 **end while**


Where: *T* is a decision table; *p* is the ceiling of then number of percentage of the selected best attributes in the formed ranking; *R* is the set of generated rules; *v* is the unique set of values from the *T* in EAV form grouped per rows of input table *T*; $v_{i}$ and $v_{i}^{\prime }$ are temporary subsets of *v* for the sake of rule generating iteration, based on the values included in the set *v* and its subsets separable subtables are created.

**Example** **1.** *This example presents work of the Algorithm 1 for decision table shown in Figure 1.*

*Percentage of best attributes which will be taken into consideration is 40%, so ceiling of the number of attributes needs to be taken is 2. The attributes standard deviations per decision class are calculated using averages’ values of attributes, for each attribute and decision value from EAVD representation, and they are the following: for $f_{1},STD=0.29$, for $f_{2},STD=0.58$, for $f_{3},STD=0.5$. It allows to create a ranking of attributes:*

- *$f_{2}\to 0.58$,*
- *$f_{3}\to 0.5$,*
- *$f_{1}\to 0.29$.*

*So the chosen best attributes are: $f_{2}$ and $f_{3}$. Standard deviations are the highest and normal distributions are the widest (see Figure 2). Value sets v of the chosen p attributes, for each row, from the decision table are the following:*

- *$f_{2},f_{3}=\left\{1,1\right\}$.*

*Separable subtable T(f2,1)*

$f_{1}$

$f_{2}$

$f_{3}$

*d*
111101021102
*is not degenerate (rows have different decisions), so subtable T(f2,1) needs to be partitioned and subtable T(f2,1)(f3,1) is obtained.*

$f_{1}$

$f_{2}$

$f_{3}$

*d*
1111
*It is degenerate table, so the rule $f_{2}=1\wedge f_{3}=1\to d=1$, associated with the first row of exemplary decision table is derived.*

- $f_{2},f_{3}=\left\{1,0\right\}$

*Separable subtable T(f2,1) is not degenerate, so subtable T(f2,1)(f3,0) is obtained.*

$f_{1}$

$f_{2}$

$f_{3}$

*d*
01021102
*It is degenerate table, so decision rule $f_{2}=1\wedge f_{3}=0\to d=2$ is derived. This rule is associated with the second and third row from exemplary decision table.*

- $f_{2},f_{3}=\left\{0,1\right\}$

*Subtable $T(f_{2},0)$ is degenerate.*

$f_{1}$

$f_{2}$

$f_{3}$

*d*
00131003
*Derived decision rule $f_{2}=0\to d=3$ is associated with the fourth and fifth row of exemplary decision table.*

- $f_{2},f_{3}=\left\{0,0\right\}$

*Subtable $T(f_{2},0)$ is degenerate and decision rule $f_{2}=0\to d=3$ is associated with the fourth and fifth row of exemplary decision table.*

*The resulting set R of decision rules derived for rows from exemplary decision table is as follows:*

- $1,f_{2}=1\wedge f_{3}=1\to d=1$
- $2,f_{2}=1\wedge f_{3}=0\to d=2$
- $3,f_{2}=1\wedge f_{3}=0\to d=2$
- $4,f_{2}=0\to d=3$
- $5,f_{2}=0\to d=3$

*When duplicated rules are removed we obtain:*

- $f_{2}=1\wedge f_{3}=1\to d=1$
- $f_{2}=1\wedge f_{3}=0\to d=2$
- $f_{2}=0\to d=3$

**Figure 3:**
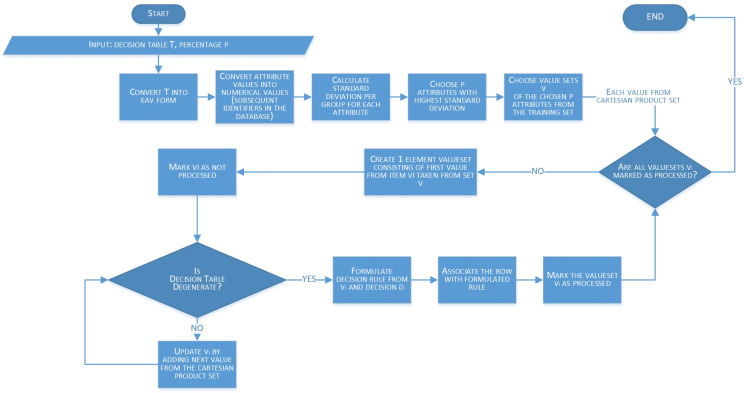
State transition diagram (STD) of the Algorithm 1.

The state transition diagram (Figure 3) visually presents the Algorithm 1 for decision rule construction.

Taking into account the state transition diagram, it is seen that the algorithm consists of a set of preprocessing steps before two main rule generating loops start to work. Two nested loops are a potential bottle neck from the computational complexity analysis point of view, but as it is explained in the next section, in an average case it is negligible. Moreover, thanks to the attribute ranking, in the most likely case scenario, algorithm can finish operation fast and generate good quality rules (which is detailed described in the Section 4).

#### 3.1.5. Algorithm Computational Complexity Analysis

In order to calculate the computational complexity of the proposed algorithm, let us analyze each of the algorithm’s steps. The steps have been rewritten from Figure 3 and gathered with their execution times in Table 1. The information is based on the assumption that *N* is the number of objects in the decision table *T*, whilst *P* is the number of input parameters, *V* is the number of valuesets $v_{i}$ and $V_{i}$ is the number of attributes’ values for a given attribute *i*.

Summing up all execution times, we get the following time complexity of the algorithm (Equation (1)):

(1) $$\begin{array}{cc} T(N) & =t_{1}+t_{2}+t_{3}+t_{4}+t_{5}+V\cdot t_{6}+V\cdot t_{7}+V\cdot t_{8}+V\cdot V_{i}\cdot t_{9}+\\ \; & +V\cdot V_{i}\cdot t_{10}+V\cdot t_{11}+V\cdot t_{12}+V\cdot t_{13}+t_{14} \end{array}$$

which can be expressed in the form (Equation (2)):

(2) $$\begin{array}{cc} T(N) & =V\cdot V_{i}\cdot \left(t_{9}+t_{10}\right)+\\ \; & +V\cdot (t_{6}+t_{7}+t_{8}+t_{10}+t_{11}+t_{12}+t_{13})+\\ \; & +t_{1}+t_{2}+t_{3}+t_{4}+t_{5}+t_{14}. \end{array}$$

Approximating the equation by introducing time constants, i.e.: $t_{9:10}=t_{9}+t_{10}$, $t_{6:8-10:13}=t_{6}+t_{7}+t_{8}+t_{10}+t_{11}+t_{12}+t_{13}$ and $t_{1:5-14}=t_{1}+t_{2}+t_{3}+t_{4}+t_{5}+t_{14}$, Equation (2) can be simplified as follows (Equation (3)):

(3) $$T\left(N\right)=V\cdot V_{i}\cdot t_{9:10}+V\cdot t_{6:8-10:13}+t_{1:5-14}.$$

Taking into account the properties mentioned earlier: 1≤V≤N and $1\leq V_{i}\leq N$, computational complexity of the algorithm can be determined:

- optimistic complexity:
  (4) $$T_{O}\left(N\right)=1$$
- average complexity—due to the fact that $V_{i}$ is typically a small constant, sometimes even equal to 2 (for binary attributes) whilst *V* is typically close to *N*, the complexity can be expressed as follows:
  (5) $$T_{A}\left(N\right)=N$$
- pessimistic (worst) complexity:
  (6) $$T_{W}\left(N\right)=N^{2}.$$

Taking into account the computational complexity of the dynamic programming approach introduced in [21], it is seen that the proposed solution is much more time efficient. Whilst for the described algorithm, the computational complexity is linear in the average scenario, for the dynamic programming approach, it could be exponential in many cases.

## 4. Results

Experiments were performed on decision tables from UCI Machine Learning Repository [45]. When for some of the decision tables, there were attributes taking unique value for each row, such attributes were removed. When some of the decision tables contained missing values, each of these values was replaced with the most common value of the corresponding attribute. When, in some of the decision tables, there were equal values of conditional attributes but different decisions, then each group of identical rows was replaced with a single row from the group with the most common decision for this group.

The aim of performed experiments was to measure quality of decision rules constructed by proposed algorithm. Decision rules were evaluated from the point of view of:

- knowledge representation, i.e., length and support of obtained rules were calculated and compared with optimal decision rules obtained by dynamic programming approach,
- knowledge discovery, i.e., classification error was calculated and compared with classifiers obtained by dynamic programming approach.

Table 2, Table 3 and Table 4 gather information on minimum, average and maximum rule length and minimum and average and maximum support of the decision rules generated by the proposed algorithm, respectively. The results were obtained for the 100%, 80% and 60% of best attributes chosen during the rule generation.

Taking into account the rule length it can be seen that for some data sets, the maximum and average values are much smaller than the number of conditional attributes in decision table, for example, lymphography, zoo-data. In case of support, maximum value should be noticed comparing to the number of rows in decision table, for cars, hayes-roth-data, house-votes and zoo-data.

The mentioned quality measures needed to be compared with the ones obtained for the optimal rules (with respect to length and support respectively) generated by the DP (dynamic programming) approach shown in [46]. In order to be able to make the comparison more informative, the relative difference of the respective results have been calculated. It is defined as follows:

(7) $$Relative\_difference=\frac{value\_for\_algorithm-value\_for\_DP}{value\_for\_DP}$$

The results are presented in Table 5, Table 6 and Table 7. Following the formula for the relative difference, it can be seen that positive values mean that results for the proposed algorithm are larger than the ones obtained for the DP approach, whilst negative values mean that results for the DP approach are larger than the ones obtained for the proposed algorithm. Zero means that both values are equal.

The presented results show that the rules generated by the proposed algorithm are not far from the optimal ones. Moreover, reducing the number of attributes resulted in an improvement of the rule quality (for 60% of attributes quality is the closest to the optimal one). Values in Table 5, Table 6 and Table 7 marked in bold denote results close to the ones obtained for the DP approach for the rules optimized with respect to their length and support respectively. It should be noticed that in Table 7 for the balance-scale decision table, the negative relative difference value is due to the fact that the number of attributes in data set was reduced to 60% of the whole number of attributes, so the average value of the constructed rules was shorter than the optimal value.

Experiments connected with classification have also been performed. To make comparison with results obtained for DP approach, the classification procedure was the same as the one described in [20].

Table 8 presents average classification error, for decision tables with 100%, 80% and 60% of best attributes, using two-fold cross validation method. For each decision table experiments were repeated 50 times. Each dataset was randomly divided randomly into three parts: train—30%, validation—20%, and test—50%. Rule-based classifier was constructed on the train part, then pruned by minimum error on validation set and used on test part of decision table. Presented classification error it is the number of objects from the test part of decision table which are incorrectly classified divided by the number of all objects in the test part of decision table. The last row of Table 8 presents the average classification error for all considered decision tables. Column Std denotes standard deviation for obtained results. The smallest mean errors have been marked in bold-the ones for which mean error is smaller than twenty percent.

The classification results obtained for the DP approach (introduced in [20]) are presented in Table 9.

Statistical analysis of classification results using the Wilcoxon two-tailed test has also been performed as per [47]. The classification results have been compared with the ones for DP approach for decision rules optimized relative to length and support respectively. For Table 8 it turns out that $min\left(W_{+},W_{-}\right)>W_{crit}$ (for 100%, 80% and 60% of best attributes), so the conclusion can be made that there is no significant difference between classification results obtained by the proposed algorithm and the DP approach. The null hypothesis has been confirmed. The goal of the proposed algorithm was to construct short rules, of enough good support, but keeping high level of decision values’ distinguishability.

All experiments were performed on a portable computer with the following technical specifications:

- Intel i5-8365U CPU,
- 16 GB of RAM memory,
- Windows 10 Enterprise x64 operating system.

The algorithm has been implemented in Java 8 accompanied by Spring Boot framework. All data related calculations have been performed on PostgreSQL 13.0 RDBMS. Software communicates with the database through JDBC connector.

## 5. Conclusions

A new algorithm for decision rules generation has been introduced in this paper. It has been shown that in the average case its computational complexity is linear. It makes the algorithm applicable to a vast majority of different data sets. Moreover, it has been experimentally shown that the rules generated by the mentioned algorithm are, of comparable classification quality to the ones generated by the dynamic programming approach which is not enough good from the time and memory complexity point of view, in opposite to the proposed one. Additionally, presented approach due to its specificity, allows to reduce number of attributes choosing the most significant ones and thus allowing to minimize computational effort even to a larger extent. Having compared the results by means of Wilcoxon test, it is possible to state that for 100%, 80% and 60% of attributes, classification results for the rules generated by the proposed algorithm are comparable to the ones obtained by the dynamic programming approach. It means that the number of attributes taken into consideration can be reduced by forty percent and still the classification results are satisfactory. Furthermore, reduction of the number of attributes often increases the support of generated decision rules and helps to avoid their over-learning.

In our future works, we would like to compare the proposed solution with other approaches for decision rules construction, e.g., heuristic-based approach. We also plan to look for further improvements and algorithm tuning. Additionally, we plan to look for its potential real-world applications.

## Figures and Tables

**Table 1:** Algorithm 1 steps with their execution times.

Step	Operation	Execution Time
1	Convert *T* into eav form	$t_{1}$
2	Convert attribute values into numerical values	$t_{2}$
3	Calculate std per decision class for each attribute	$t_{3}$
4	Choose *p* attributes with highest std value	$t_{4}$
5	Choose sets *v* of the chosen *p* attributes from the training set	$t_{5}$
6	Are all valuesets $v_{i}$ marked as processed	$V\cdot t_{6}$, where 1≤V≤N
7	Create 1 element valueset consisting of first value from item $v_{i}$ taken from set *v*	$V\cdot t_{7}$, where 1≤V≤N
8	Mark $v_{i}$ as not processed	$V\cdot t_{8}$, where 1≤V≤N
9	Does the corresponding subtable is degeneare?	$V\cdot V_{i}\cdot t_{9}$, where 1≤V≤N and $1\leq V_{i}\leq N$
10	Update $v_{i}$ by adding next value from the cartesian product set	$V\cdot V_{i}\cdot t_{10}$, where 1≤V≤N and $1\leq V_{i}\leq N$
11	Formulate decision rule from $v_{i}$ and the most common decision *d*	$V\cdot t_{11}$, where 1≤V≤N
12	Associate the row with the formulated rule	$V\cdot t_{12}$, where 1≤V≤N
13	Mark the valueset $v_{i}$ as processed	$V\cdot t_{13}$, where 1≤V≤N
14	END	$t_{14}$

**Table 2:** Rule-quality measures for 100% attributes.

Decision Table	Number of		Rule Length			Rule Support		
	Rows	Attributes	Min	Avg	Max	Min	Avg	Max
balance-scale	625	4	3	3.64	4	1	2.44	5
breast-cancer	266	9	1	5.40	9	1	3.72	22
cars	1728	6	1	3.61	6	1	207.01	576
hayes-roth-data	69	5	1	2.64	4	1	3.81	12
house-votes	279	16	3	6.61	16	1	30.73	82
lymphography	148	18	1	3.93	12	1	2.70	9
nursery	12,960	8	7	7.14	8	1	2.72	3
shuttle-landing-control	15	6	1	3.07	6	1	1.93	3
soybean-small	47	35	2	4.40	9	1	2.79	5
zoo-data	59	16	1	4.86	10	1	5.61	12

**Table 3:** Rule-quality measures for 80% of best attributes.

Decision Table	Number of		Rule Length			Rule Support		
	Rows	Attributes	Min	Avg	Max	Min	Avg	Max
balance-scale	625	4	3	3.64	4	1	2.44	5
breast-cancer	266	9	1	5.32	8	1	3.72	22
cars	1728	6	1	3.20	5	2	207.55	576
hayes-roth-data	69	5	1	2.64	4	1	3.81	12
house-votes	279	16	3	6.69	13	1	30.73	82
lymphography	148	18	1	3.93	12	1	2.70	9
nursery	12,960	8	7	7.00	7	2	2.86	3
shuttle-landing-control	15	6	1	2.58	5	1	1.93	3
soybean-small	47	35	2	4.40	9	1	2.79	5
zoo-data	59	16	1	4.41	10	1	5.61	12

**Table 4:** Rule-quality measures for 60% of best attributes.

Decision Table	Number of		Rule Length			Rule Support		
	Rows	Attributes	Min	Avg	Max	Min	Avg	Max
balance-scale	625	4	3	3.00	3	2	3.97	5
breast-cancer	266	9	1	4.83	6	1	4.12	22
cars	1728	6	1	2.78	4	6	210.46	576
hayes-roth-data	69	5	1	2.33	3	1	3.94	12
house-votes	279	16	3	6.21	10	1	30.99	82
lymphography	148	18	1	3.87	11	1	2.70	9
nursery	12,960	8	5	5.00	5	8	10.17	12
shuttle-landing-control	15	6	1	2.36	4	1	1.93	3
soybean-small	47	35	2	4.40	9	1	2.79	5
zoo-data	59	16	1	4.46	10	1	5.61	12

**Table 5:** Relative difference of length and support of decision rules for 100% attributes.

Decision Table	Average Rule Length	Average Rule Support
balance-scale	**0.14**	**−0.42**
breast-cancer	1.03	−0.61
cars	**0.48**	**−0.38**
hayes-roth-data	**0.23**	**−0.42**
house-votes	1.60	−0.58
lymphography	0.97	−0.87
nursery	1.29	−1.00
shuttle-landing-control	1.19	**−0.09**
soybean-small	3.40	−0.78
zoo-data	2.12	−0.49

**Table 6:** Relative difference of length and support of decision rules for 80% attributes.

Decision Table	Average Rule Length	Average Rule Support
balance-scale	**0.14**	**−0.42**
breast-cancer	1.00	−0.61
cars	**0.32**	**−0.38**
hayes-roth-data	**0.23**	**−0.42**
house-votes	1.60	−0.58
lymphography	0.97	−0.87
nursery	1.25	−1.00
shuttle-landing-control	0.85	**−0.09**
soybean-small	3.40	−0.78
zoo-data	1.83	−0.49

**Table 7:** Relative difference of length and support of decision rules for 60% attributes.

Decision Table	Average Rule Length	Average Rule Support
balance-scale	**−0.06**	**−0.07**
breast-cancer	0.81	−0.57
cars	**0.14**	**−0.37**
hayes-roth-data	**0.09**	−0.40
house-votes	1.45	−0.58
lymphography	0.94	−0.87
nursery	0.60	−0.99
shuttle-landing-control	0.69	**−0.09**
soybean-small	3.40	−0.78
zoo-data	1.86	−0.49

**Table 8:** Average classification error for the proposed algorithm.

Decision Table	Number of		100% of Attributes		80% of Attributes		60% of Attributes	
	Rows	Attributes	Mean Error	Std	Mean Error	Std	Mean Error	Std
balance-scale	625	4	0.45	0.08	0.45	0.08	0.48	0.08
breast-cancer	266	9	**0.00**	0.00	**0.00**	0.00	**0.00**	0.02
cars	1728	6	**0.07**	0.11	**0.08**	0.13	**0.18**	0.21
hayes-roth-data	69	5	0.52	0.10	0.52	0.10	0.51	0.12
house-votes	279	16	0.25	0.12	0.25	0.12	0.26	0.14
lymphography	148	18	**0.16**	0.11	**0.16**	0.10	**0.14**	0.11
nursery	12,960	8	**0.00**	0.01	**0.00**	0.01	**0.00**	0.0
shuttle-landing-control	15	6	0.22	0.19	**0.18**	0.17	0.22	0.18
soybean-small	47	35	0.21	0.14	0.21	0.14	0.21	0.14
zoo-data	59	16	0.40	0.26	0.38	0.25	0.36	0.24
average	-	-	0.21	0.10	0.20	0.10	0.21	0.11

**Table 9:** Average classification error for the DP approach.

Decision Table	DP Optimized w/r Length	DP Optimized w/r Support
	Mean Error	Mean Error
balance-scale	0.29	0.28
breast-cancer	0.31	0.30
cars	0.22	0.21
hayes-roth-data	0.37	0.35
house-votes	0.08	0.05
lymphography	0.35	0.28
nursery	0.05	0.05
shuttle-landing-control	0.40	0.39
soybean-small	0.17	0.17
zoo-data	0.24	0.18
average	0.25	0.23

## Data Availability

Publicly available datasets were analyzed in this study. This data can be found here: [45].

## Abbreviations

The following abbreviations are used in this manuscript:

EAV	Entity–Attribute–Value
EAVD	Entity–Attribute–Value-Decision
DP	Dynamic Programming
STD	Standard Deviation
